# Fluorescent Labeling Methods for Brain Structure Research

**DOI:** 10.3390/molecules31050817

**Published:** 2026-02-28

**Authors:** Chunguang Yin, Jiangcan Li, Keyu Meng, Jiade Zhang, Meihe Chen, Ruibing Chen, Yuyang Hu, Shuodong Wang, Sheng Xie

**Affiliations:** 1 State Key Laboratory of Chemo and Biosensing, College of Chemistry and Chemical Engineering, Hunan University, Changsha 410082, China; 2 Shenzhen Research Institute of Hunan University, Nanshan District, Shenzhen 518000, China

**Keywords:** fluorescent labeling, neural circuits, viral tracing, immunofluorescence, brain imaging

## Abstract

The brain is a complex structural network. The employment of fluorescent labeling techniques in conjunction with advanced imaging methodologies facilitates comprehensive analysis of multiscale brain anatomy, thereby offering insights into fundamental principles of function and addressing neurological disorders. This review summarizes technological advances in fluorescent labeling methods in the field of neuroscience, and their applications in neural circuit analysis, cerebrovascular imaging, neuronal activity monitoring, and fluorescence-guided treatment of brain tumors. A challenging trend in integrating smart fluorescent labeling with tissue clearing, wide-field 3D imaging, artificial intelligence-assisted data processing/reconstruction, and multimodal information fusion is highlighted and discussed. The future direction of combining high-resolution, low-damage, dynamic imaging with big data analysis is envisioned, providing tools for understanding brain structure and function and their roles in disease.

## 1. Introduction

The human brain, one of the most complex systems in the known universe, comprises a sophisticated network of nearly 86 billion neurons and trillions of synapses, serving as the physical basis for all thoughts, emotions, and behaviors [1]. Fully deciphering the brain’s structural connectome is not only an ultimate goal of neuroscience but also a cornerstone for understanding brain function principles and addressing neurological disorders [2]. However, this exploration faces unprecedented intrinsic challenges. The primary challenge stems from the brain’s inherent extreme complexity and multiscale nature, with its organizational structure spanning vast spatial dimensions—from nanoscale (e.g., synaptic clefts and vesicles), to microscale (e.g., neuronal morphology and axonal projections), to macroscale (e.g., whole-brain circuits) [3]. This implies that no single research technique can penetrate all scales, resulting in an observational ‘scale gap’ [4,5]. Additionally, understanding brain structure requires more than static mapping. It necessitates long-term observation of dynamic processes. However, traditional invasive methods often involve significant phototoxicity and photobleaching, failing to meet the need for long-term, stable monitoring of neural plasticity in living organisms [6]. Lastly, with advances in imaging technology, data processing has become a new bottleneck. The terabyte- to petabyte-scale image data poses immense computational challenges for automated, intelligent neuronal reconstruction, segmentation, and analysis [7]. These challenges underscore the urgent need for a revolutionary research paradigm capable of achieving multiscale, high-resolution, low-damage, dynamic imaging that is compatible with intelligent big data analysis [8].

Clinical imaging techniques have made significant contributions to the study of brain structure [9]. The core issues include insufficient spatial resolution (MRI and PET) or poor soft tissue contrast (CT), which fail to meet the precision required for resolving micron-scale cellular and circuit structures. Consequently, these techniques are primarily used for macroscopic screening and longitudinal tracking in vivo, rather than fine structural analysis [10]. The emergence and development of fluorescence labeling techniques have become a critical key and driving force in unraveling the mysteries of brain structure [11]. By enabling targeted expression of specific fluorescent reporter molecules (e.g., green fluorescent protein GFP) in specific cell types, neural circuits, or organelles, this technology allows researchers to “optically label” and “genetically target” brain components with unprecedented specificity and clarity, transforming invisible neural structures into visible optical signals. This not only greatly enhances our understanding of brain cellular architecture but also, more importantly, provides an indispensable source of imaging contrast for advanced imaging techniques.

In recent years, fluorescence labeling techniques have achieved significant improvements in labeling efficiency, signal stability, and biocompatibility by integrating chemical synthesis, gene editing, viral engineering, and nanotechnology. Small-molecule organic probes, with their high affinity and cell-penetrating capabilities, can directly label endogenous targets [12]. Genetic engineering approaches enable precise integration of fluorescent proteins, peptides, or even single amino acids into target proteins for specific labeling and dynamic tracking [13]. Immunostaining techniques leverage the high specificity of antibodies (primarily IgG) to visualize target proteins through direct (fluorophore-conjugated primary antibodies) or indirect (fluorophore-conjugated secondary antibodies) methods [14]. Additionally, fluorescently engineered viral vectors can infect neurons and propagate and replicate within them, thereby enabling effective axonal or trans-synaptic neural circuit tracing [15]. Progress has advanced from random expression in specific transgenic animals to cell-type-specific expression based on the Cre-loxP system [16], and further to flexible and efficient site-specific labeling mediated by viral vectors [17]. Techniques such as Brainbow and FLARE [18] have enabled multicolor labeling, which allows for the parallel tracking of multiple neural pathways within the same sample. In particular, CRISPR/Cas9-mediated labeling of genes has made it possible to study protein distribution and function at physiological expression levels [19]. Combining tissue clearing techniques with fluorogenic labeling overcomes tissue opacity and scale barriers, making spatial reconstruction of structural dynamics achievable [20,21]. Thus, fluorescence labeling technology is not an isolated tool but a bridge connecting molecular biology (labeling strategies) [22], optical imaging (observation methods) [23], and computational science (data analysis) [24], serving as a core engine in advancing brain science [25,26].

**Figure 1:**
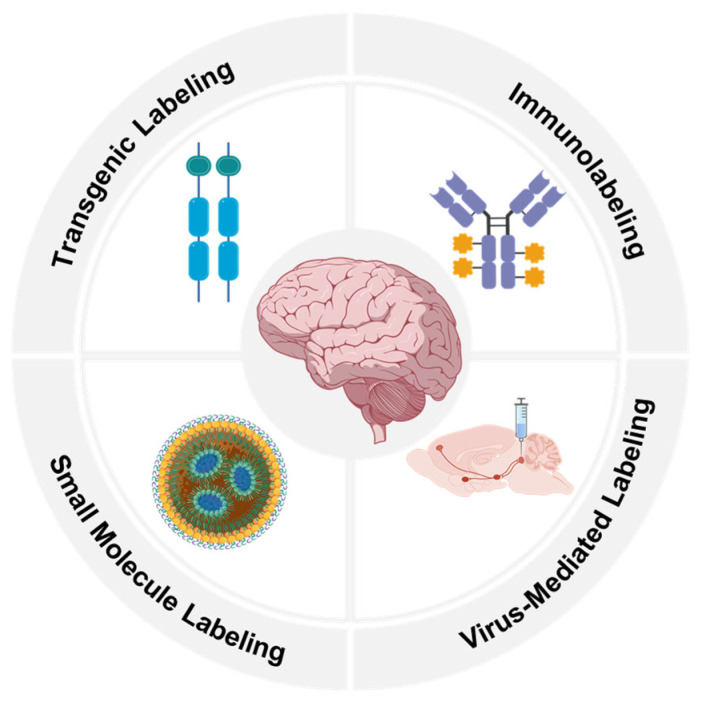
Schematic overview of key neuroscience research focuses relevant to fluorescent labeling applications: small molecule labeling, transgenic labeling, immunolabeling and virus-mediated labeling.

This review outlines the technological evolution of fluorescence labeling methods and details how they integrate with advanced imaging techniques to overcome challenges in brain structure research (Figure 1). The review also looks forward to future directions, including higher-performance probes, less invasive in vivo labeling strategies, and data analysis paradigms integrated with artificial intelligence. To facilitate comparison of the core features and applicable scenarios of the various fluorescent labeling methods described above, we have summarized them in Table 1. This table systematically compares the major techniques across key dimensions, including specificity, toxicity, penetration depth, long-term stability, and reproducibility, serving as a quick reference for researchers in selecting appropriate labeling strategies [27,28,29,30,31,32,33].

## 2. Neural Circuit Labeling and Imaging

The brain is made up of dense circuit-like networks of interconnected neurons. Neural circuits form the basis of brain function. Electron microscopy (EM) can provide synaptic resolution and is used as a gold standard in connectomics [34]. However, due to its extremely large data size and imaging process, the reconstruction process is the bottleneck and it is now typically possible to obtain EM images only for 1 mm^3^ volumes (~petabyte scale) [35,36]. A fundamental task in neuroscience and neurology is mapping structural connectivity among different brain regions and neurons of the brain, ideally at subcellular resolution. Fluorescence imaging has been a powerful tool for visualizing the three-dimensional structure of neuronal morphology and mesoscopic connectivity, even for the mesoscopic circuit mapping at a whole-brain level [37].

### 2.1. Lipophilic Labeling of the Neuronal Membrane

**Figure 2:**
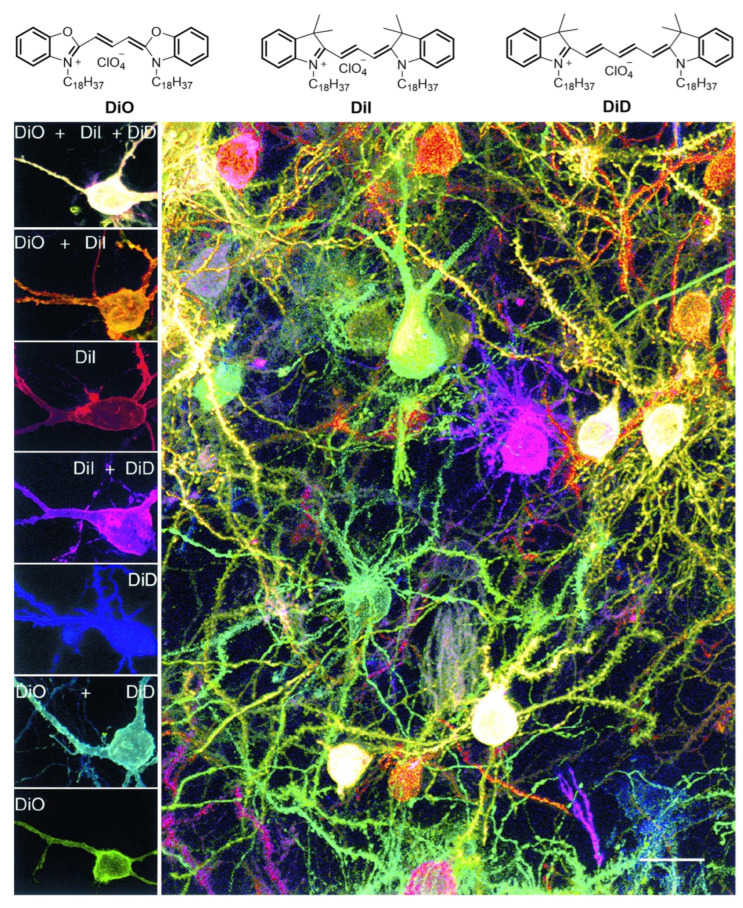
DiOlistic labeling of neuron membrane structures and circuits. Adapted with permission from reference [45]. Copyright 2000 Neuron.

Lipophilic neuron membrane labeling is a well-established technique for the labeling of neurons in neural circuit research. It relies on lipophilic dyes that are able to diffuse across cell membranes, thereby enabling rapid and uniform labeling of neuronal cell membranes. Since their initial application in neuroscience in the 1990s, the types and functions of lipophilic dyes have undergone continuous expansion, and they have become crucial tools for neural circuit imaging [38]. Lipophilic neuronal membrane labeling can unveil the dynamic architecture of neuronal morphology, axonal projections and synaptic connections, thereby elucidating circuit functions and their behavioral correlates. In neural circuit research, this method visualizes the membrane lipid bilayer structure, compensating for the temporal constraints of genetic labeling to enable rapid, non-invasive tracking, particularly suited for developmental and injury models [39]. The underlying principle relies on the hydrophobic insertion of lipophilic dyes into the cell membrane lipid bilayer, followed by passive diffusion along the membrane surface to achieve long-distance tracing [40]. Common membrane-specific fluorophores include carbocyanine dyes (e.g., DiI and DiO) [41] and styryl dyes (e.g., FM1-43) [42], which can be applied via microinjection or immersion and are compatible with both fixed and live tissues. Among these, switchable “on–off” fluorescent molecules such as spiropyran have been employed in single-molecule localization microscopy techniques including stochastic optical reconstruction microscopy (STORM) and photoactivated localization microscopy (PALM), enabling temporal separation of individual emitter signals and achieving nanometer-scale resolution [43,44]. In neural circuit labeling applications, Lichtman et al. utilized particle-mediated ballistic delivery of lipophilic dyes (DiO, DiI and DiD) to differentially label cells across a range of neuronal tissues (Figure 2) [45]. In vivo, neurons that had been labeled exhibited normal synaptic responses and underwent dendritic remodeling. Despite its pronounced advantages, this method remains constrained by non-specific labeling, diffusion-induced artefacts, and photobleaching, which collectively limit its utility in deep-tissue applications.

With the integration of super-resolution microscopy and tissue clearing techniques, these membrane-specific dyes now enable subcellular-resolution mapping of membrane structures in large brain volumes [46]. Recent advances, including membrane-anchored fluorescent proteins, click chemistry-based lipid conjugates, and photoactivatable membrane probes have improved both temporal control and spatial resolution [47,48,49]. Lipid-based labeling of neuronal membranes is now widely used to delineate neuronal morphology, trace long-range axonal projections and reconstruct 3D connectomes.

### 2.2. Silver-Mediated Labeling of Argyrophilic Neural Structures

Silver stains are regarded as the gold standard in histological brain staining [50,51]. The first method by Camillo Golgi in the 1870s, and the subsequent Cajal’s stain by Santiago Ramón y Cajal, exploits the special affinity of ionic silver species towards neural structures and the reduction-based chromogenic silvering reaction into metallic silver grains [52,53]. These processes collectively result in the staining of the sample dark brown or black. It has been observed that only ~1% of neurons can be labeled by the Golgi silver stain. However, this small subset of stained cells offers great detail on neuron morphology due to the sparsity of the stain [54].

**Figure 3:**
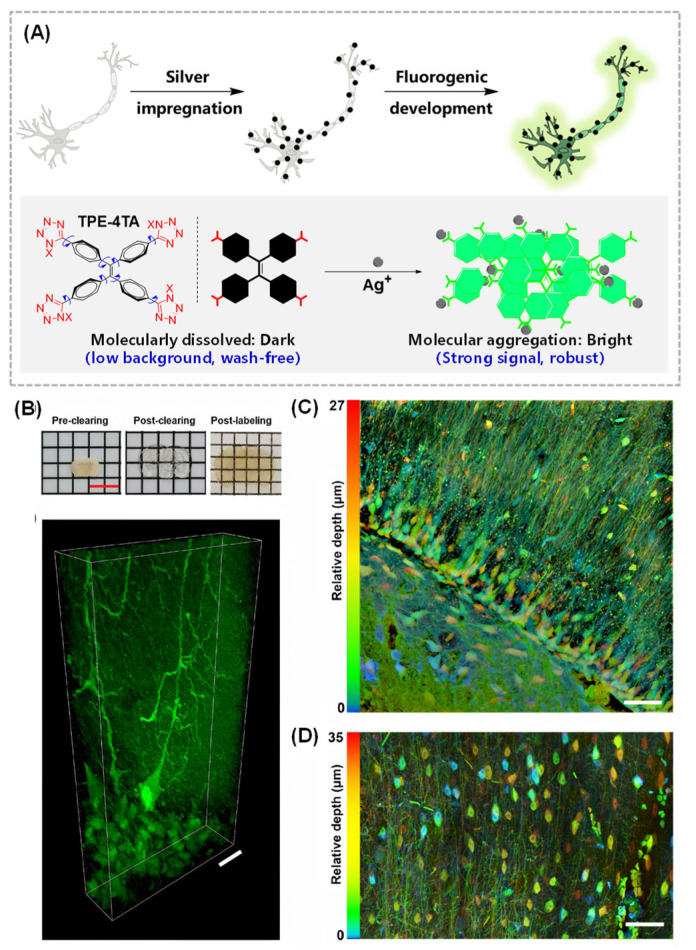
Silver-AIE labeling of multiple nervous structures. (**A**) Schematic illustration of development in the silver-AIE method. (**B**) Top: Photograph of pre-clearing, post-clearing and post-labelling of a 1-mm-thickcoronal section of a mouse brain, brightness adjusted to match. Scale bar: 12 mm. Bottom: 3D rendering of a silver-AIE stained neuron in the mouse cerebellum with a depth of 39.6 μm imaged with a 60×/1.2 NA water immersion objective lens on a confocal microscope. Scale bar: 20 μm. (**C**) Confocal image of the hippocampus from the CLARITY-cleared mouse brain coronal section, depth colour-coded z-stack projection from 0 to 27 μm HDR image with shading correction and denoised with a gaussian filter imaged using a 20×/0.8 NA objective lens. Scale bar: 50 μm. Dimensions: 200 μm length × 200 μm width × 27 μm depth. (**D**) Depth colour-coded cropped tile scan with a 35 μm depth range of the CLARITY-cleared cortex using a 63×/1.4 NA oil immersion objective lens. Adapted with permission from reference [55]. Copyright 2025 SCIENCE CHINA Chemistry.

Xie et al. reported a fluorogenic silver-based brain staining method, named silver-AIE, which utilized an Ag^+^-triggered aggregation-induced emission (AIE) labeling process to visualize the argyrophilic structure in the brain tissue (Figure 3) [55]. This method involves the in situ self-assembly of fluorogenic metal–coordination polymers, which enables highly efficient signal amplification and wash-free staining (Figure 3A). Ionic silver species, which bind to the argyrophilic chemical groups in proteins and other macromolecules, can be fluorescently developed using the water-soluble TPE-4TA probe [56]. This method surpasses the chromogenic detection step and eliminates the associated high background, artefacts, and poor reproducibility in traditional silver stains. The silver-AIE staining was applied in staining the hydrogel-based CLARITY-cleared mouse brain (Figure 3B). A range of characteristic nervous structures, including the hippocampal neurons, white matter tracts and Purkinje cells, could be observed clearly with a high contrast in 1 mm-thick cleared tissue slices. A multipolar neuron with an axon of >150 µm long was observed in the cerebellum, suggesting the potential in tracing neuronal connectivity (Figure 3B). The delicate arrays of the axonal fibers were revealed in the z-stack projection of confocal images in the hippocampal CA region (Figure 3C). The cell networks with the axonal fibers in the CLARITY-cleared cortex could be observed clearly in the depth color-coded tile scan (Figure 3D). This modernized silver-AIE staining technique has revitalized the traditional method and provided rich opportunities for systematic neuropathological analysis.

### 2.3. Immunofluorescence Labeling of Specific Targets in Neural Circuits

Immunofluorescence uses fluorophore-conjugated antibodies to detect target molecules. Immunofluorescence labeling stands as a cornerstone in neural circuit imaging, prized for its molecular precision in targeting membrane proteins, synaptic markers and circuit elements [57,58]. This illuminates the structure–function relationships and pathological alterations. In neural circuit research, immunofluorescence offers epitope-specific visualization that can dissect local networks to global connectomes [59]. The principle hinges on antigen–antibody affinity: primary antibodies bind to targets such as neuronal membrane antigens [60] or synaptic scaffolds [61], followed by direct (fluorophore-conjugated primaries) or indirect (secondary amplification) signal transduction. Since its development in the 1950s, immunofluorescence technology has continuously evolved, with fluorophores advancing from early examples such as FITC (fluorescein isothiocyanate) to the modern Alexa Fluor and Cy series dyes, which offer improved photostability and broader spectral ranges [62,63,64]. When compared with radioactive or enzyme-based labeling methods, immunofluorescence provides substantial advantages in terms of spatial resolution, labeling efficiency, and experimental flexibility for neural circuit labeling and live imaging. Recent advancements in this field have given rise to nanobody-based labeling, DNA-barcoded antibodies, and multiplexed immunostaining protocols (e.g., CODEX, MIBI, and IBEX), which allow simultaneous detection of dozens to hundreds of molecular targets in large-scale brain samples [65,66,67]. Moreover, the integration of immunolabeling with functional reporters (e.g., calcium or voltage indicators) serves to link structural and activity-based circuit analyses [68,69].

**Figure 4:**
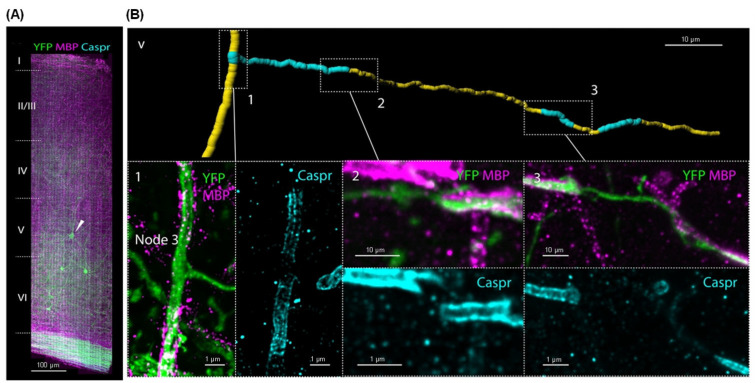
Imaging analysis of immunofluorescence-labeled neural structures through a combination of expansion microscopy and lattice light-sheet microscopy techniques. (**A**) Immunostained against MBP and Caspr. (**B**) (Top) Segmented view of a collateral axon with myelinated and unmyelinated sections from boxed region v. (Bottom) Three MIP views of breaks in myelination with flanking Caspr. Adapted with permission from reference [75]. Copyright 2019 Science.

Immunofluorescence is extensively used to reveal the connections between neurons, synaptic functions, and the dynamic changes in neural circuits [70]. By using specific antibodies to label neurons or specific synaptic proteins in particular brain regions, researchers can identify the role of different neural circuits in processes like behavior, learning, and memory [71]. For example, labeling proteins associated with synaptic plasticity, such as PSD-95 and Synaptophysin, allows spatial localization and functional analysis of neural networks [72]. The combination of immunofluorescence with advanced imaging modalities has resulted in significant advancements in imaging depth and resolution, enabling the visualization of fine synaptic details at the single-cell and even single-molecule level [73,74]. Betzig et al. employed a combination of expansion microscopy and lattice light-sheet microscopy to image the nanoscale spatial relationships between proteins across the mouse cortex, showing synaptic proteins at dendritic spines and myelination along collateral axons (Figure 4) [75]. The technology should facilitate statistically robust, large-scale studies of neural development, sexual dimorphism, degree of stereotypy, and structural correlations to behavior or neural activity, all with molecular contrast. Although immunofluorescence techniques offer unparalleled advantages for protein-specific visualization, their reliance on fixed tissue, limited penetration depth, and lack of dynamic tracking capabilities similarly hinder the advancement of large-scale real-time neural circuit analysis.

### 2.4. Virus-Mediated Labeling and Analysis of the Neural Circuit

Viral-mediated tracing is a powerful method for neural circuit labeling, utilizing the natural infectivity of viruses to transfer genetic information into specific neurons [76]. This method typically employs adenoviruses (AAVs) or retroviruses (RCVs) as vectors, delivering gene fragments that encode fluorescent proteins or enzyme markers (such as β-galactosidase) into neurons, enabling the visualization of neurons and their synaptic connections [77,78]. In circuit tracing, viruses are typically injected into specific regions of the brain, where they retrogradely or anterogradely label surrounding neurons and their synaptic networks. Since the 1990s, viral-mediated tracing has become an indispensable tool in neuroscience for mapping neural circuits with high specificity and efficiency. AAVs are favored for their low immunogenicity and ability to transduce both dividing and non-dividing cells, making them suitable for long-term expression studies [79]. Lentiviruses, with their ability to integrate into the host genome, provide stable expression in a wide range of cell types [80]. Rabies viruses are utilized for monosynaptic tracing, allowing the identification of direct presynaptic inputs to a targeted neuron population [81].

**Figure 5:**
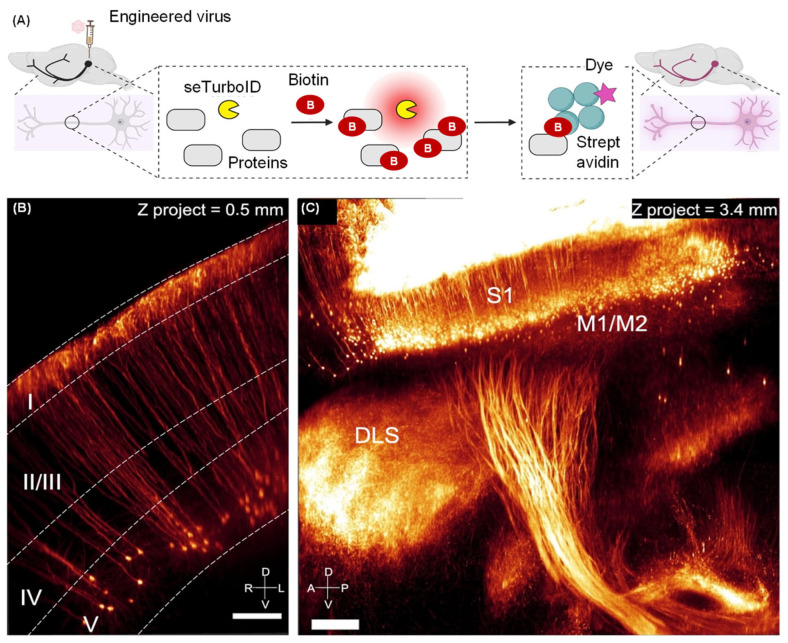
Virus-mediated fluorescent imaging of axons via the LINCS labeling system. (**A**) Schematic showing the neuronal labeling through Cre-dependent seTurboID expression, proximity biotinylation, and streptavidin staining. (**B**) Coronal light-sheet microscopy image of labeled corticospinal neurons in S2 cortex. Scale bar, 100 μm. (**C**) Image of labeled corticospinal neurons projecting from M1/M2 and S1 cortices. Scale bar: 250 μm. Adapted with permission from reference [87]. Copyright 2025 Neuron.

These viral vectors can be further engineered to express specific proteins, optogenetic tools, or calcium indicators, facilitating the visualization and manipulation of neural circuits in vivo [82,83,84]. For instance, monosynaptic rabies virus tracing enables the identification of direct presynaptic inputs to specific neuron populations, providing insights into the connectivity and functional architecture of neural circuits [85]. Furthermore, innovations such as the AAV-assisted retrograde tracing system have facilitated brain-wide labeling of genetically defined projection neurons, thereby circumventing the limitations by viral tropism and enabling comprehensive mapping of neural networks [86]. These advancements have expanded the scope of viral tracing applications and enabled more detailed and comprehensive analyses of neural circuits. Lin et al. reported an engineered virus-mediated indirect approach (named LINCS) to label individual neurons with controllable sparseness throughout the entire mouse brain and body. Following the integration of the seTurboID tag, LINCS employed the solubility-enhanced biotin ligase for in vivo biotinylation, subsequently accompanied by rapid whole-mount staining with high-affinity streptavidin (Figure 5A) [87]. When integrated with tissue clearing and light-sheet microscopy, this system creates an efficient pipeline for profiling long-range neuronal projections across both the central and peripheral nervous systems (Figure 5B,C). This integrated toolkit provides a robust and scalable pipeline for mapping neuronal connectivity, poised to accelerate the anatomical and mechanistic investigation of neural circuits.

**Figure 6:**
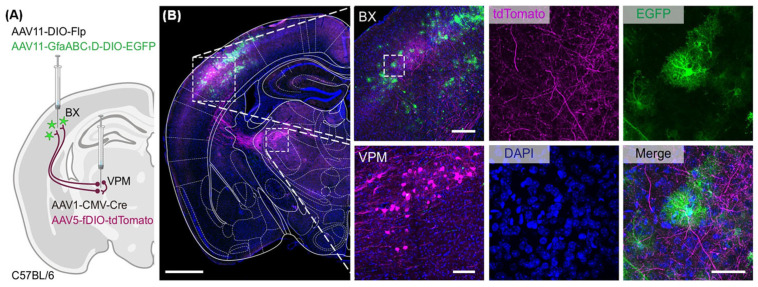
AAV-based retrograde labeling for analyzing neuron–astrocyte connections. (**A**) AAV1-CMV-Cre and AAV5-EF1α-fDIO-tdTomato were coinjected into the VPM, and AAV11-GfaABC1D-DIO-EGFP and AAV11-EF1α-DIO-Flp were coinjected into BX. (**B**) Representative images of Cre-dependent EGFP (green) labeling of BX astrocytes and tdTomato (magenta) labeling of VPM neurons, 3 weeks after AAV injection. Adapted with permission from reference [88]. Copyright 2023 nature communications.

Viral tracers that facilitate efficient retrograde labeling of projection neurons represent a powerful tool for the structural and functional dissection of neural circuits and for the treatment of brain diseases. At present, recombinant adeno-associated viruses (rAAVs) based on capsid engineering are utilized for retrograde tracing, but they usually display undesirable selectivity due to inefficient retrograde transduction in specific neural connections. Xu et al. developed a toolkit that is characterized by ease of editing and the capacity to produce high titer AAV11. The toolkit demonstrated potent and stringent retrograde labeling of projection neurons in mice (Figure 6) [88]. The GfaABC1D promoter embedding of AAV11 has been demonstrated to exhibit superior astrocytic tropism in vivo in comparison to AAV8 and AAV5 (Figure 6A). In conjunction with bidirectional multi-vector axoastrocytic labeling, AAV11 was utilized to study neuron–astrocyte connections (Figure 6B). These properties render AAV11 a promising tool for mapping and manipulating neural circuits and for gene therapy.

**Figure 7:**
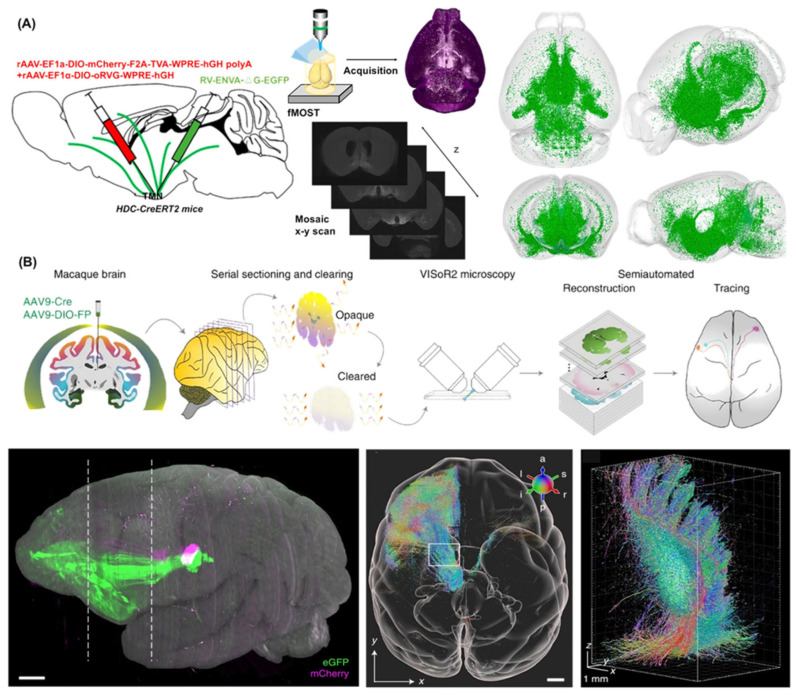
Whole-brain 3D reconstruction and visualization of long-range monosynaptic inputs to histaminergic neurons. (**A**) Whole-brain analysis of long-range monosynaptic inputs to histaminergic neurons in the neuronal histaminergic system. Adapted with permission from reference [99]. Copyright 2025 Nature Communications. The SMART approach for high-throughput mapping of a rhesus macaque brain at micron resolution. (**B**) Mesoscopic mapping of the medial dorsal nucleus of the thalamus projection using the SMART approach. Scale bar: 5 mm. Adapted with permission from reference [103]. Copyright 2021 nature biotechnology.

The neuronal histaminergic system plays a crucial role in physiological functions [89,90,91,92]. Dysregulation or lesions of this system contribute to the pathogenesis of numerous neurological disorders, including insomnia and narcolepsy, Alzheimer’s disease, Parkinson’s disease, depression and schizophrenia [93,94,95,96,97,98]. However, the structural and functional characteristics of input circuits targeting histaminergic neurons remain poorly understood. Chen et al. construct a 3D monosynaptic long-range input atlas of male mouse histaminergic neurons by integrating a fluorescence micro-optical sectioning tomography (fMOST) system with HDC-CreERT2 mice and a retrograde RV tracing system (Figure 7A) [99]. This study provided a precise long-range input map of mouse histaminergic neurons on a mesoscopic scale, laying a solid foundation for the systematic future study of histaminergic neural circuits.

Whole-brain mesoscale mapping in primates has been hindered by large brain sizes and the relatively low throughput of available microscopy methods [100,101,102]. Bi et al. developed an efficient method for long-range tracing of sparse axonal fibers in monkey brains. This pipeline, termed serial sectioning and clearing, three-dimensional microscopy with semiautomated reconstruction and tracing (SMART), enabled connectome-scale mapping of large primate brains. Utilizing the SMART approach, the authors successfully constructed a cortical projection map of the mediodorsal nucleus of the thalamus and were able to discern unique turning and routing patterns of individual axons within the cortical folds (Figure 7B) [103].

### 2.5. Transgenic Multicolor Labeling

Transgenic multicolor labeling technology is a powerful tool based on genetic engineering, widely used in neural circuit research. The Brainbow system, based on the Cre/loxP recombination system, uses Cre recombinase to randomly activate the expression of multiple fluorescent proteins (such as GFP, RFP, YFP, and CFP), enabling multicolor labeling of individual neurons and forming a unique “rainbow” visualization tool for neural circuits [104]. The Tetbow system is a further development of Brainbow, utilizing a tetracycline-inducible flip-flop system (Tet-ON/Tet-OFF) to control gene expression, allowing more flexible regulation of the number and timing of labeled neurons. These multicolor labeling systems allow researchers to distinguish and track thousands of neurons in the same region or different brain areas, providing richer neural circuit information compared to traditional single-color labeling methods [105]. The Brainbow system is particularly suitable for precise labeling of multineuronal connections in areas like the cortex and hippocampus, revealing complex neuronal connectivity, functional organization of neuronal populations, and the roles of different neural circuits in behavior [106]. In recent years, the combination of high-resolution confocal microscopy (LSCM), multiphoton microscopy (MPM), and light-sheet microscopy has made large-scale neural circuit imaging possible. The temporal controllability of the Tetbow system in recent studies allows researchers to label and observe changes in neural circuits at different time points, greatly advancing research in neural development and neuroplasticity.

**Figure 8:**
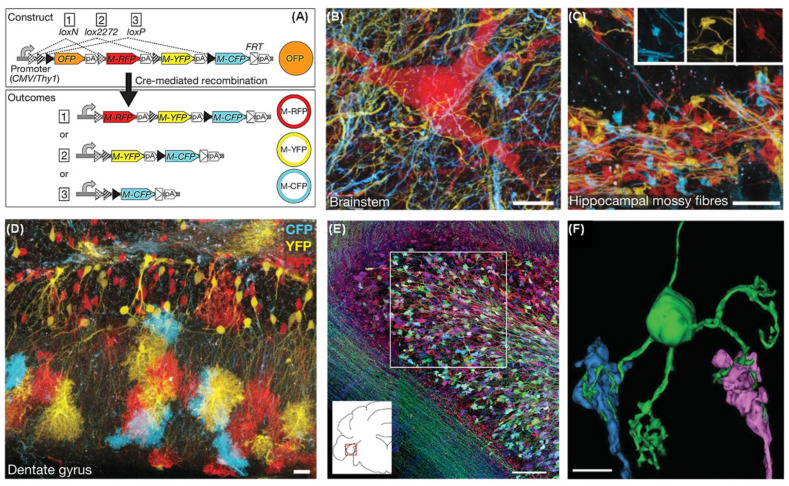
Transgenic strategies for combinatorial expression of fluorescent proteins in the nervous system. (**A**) In Brainbow-1.1, a third set of incompatible lox sites (loxN) is added, creating three recombination possibilities (1, 2 or 3), switching OFP expression to RFP, YFP or CFP expression. (**B**,**C**) Thy1-Brainbow-1.0 and Thy1-Brainbow-1.1 transgenic mice were crossed with CreERT2-expressing animals. Tamoxifen injection led to mosaic XFP expression throughout the brain. (**B**) Brainstem, line H; Scale bar: 10 μm. (**C**) Hippocampal mossy fiber axons and their terminals (see insets), line M. Scale bar: 10 μm. (**D**) Hippocampus (dentate gyrus), line Q (labeled neurons and astrocytes). (**E**) Cerebellar flocculus from line H. Inset shows coronal location. Scale bar: 50 μm. (**F**) Reconstructed granule cell receives input from ≥3 different mossy fibers (blue, pink and at least one unlabeled). Scale bar: 5 μm. Reproduced with permission from reference [107]. Copyright 2007 Nature.

Detailed analysis of neuronal network architecture requires the development of new methods. Lichtman et al. present strategies to visualize synaptic circuits by genetically labeling neurons with multiple, distinct colors. In Brainbow transgenes, the Cre/lox recombination is used to create a stochastic choice of expression between three or more fluorescent proteins (XFPs) (Figure 8A) [107]. Integration of tandem Brainbow copies in transgenic mice yielded combinatorial XFP expression, and thus many colors, thereby providing a way to distinguish adjacent neurons and visualize other cellular interactions (Figure 8B,C). The expression in some lines also allowed us to map glial territories and follow glial cells and neurons over time in vivo (Figure 8D–F). The ability of the Brainbow system to label uniquely many individual cells within a population may facilitate the analysis of neuronal circuitry on a large scale.

**Figure 9:**
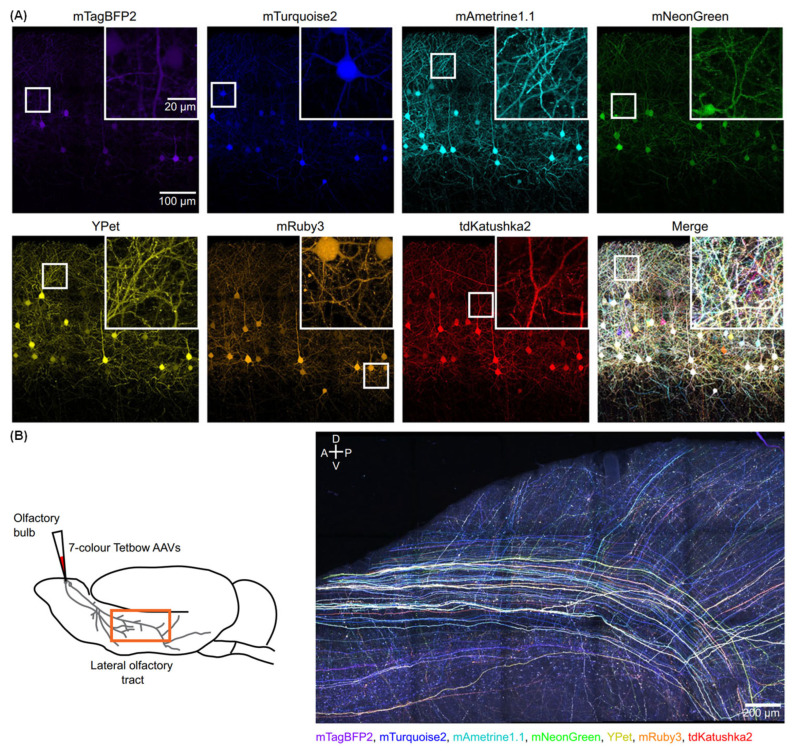
Fluorescent Tetbow labeling for brain imaging. (**A**) Layer 2/3 neurons in S1 were labeled with seven-color Tetbow using in utero electroporation. Age, postnatal day (P) 28. XFP images after linear unmixing are shown. Z-stacked images of 514  ×  513  ×  43 μm^3^. Inset displays magnified images (white box). (**B**) A 3  ×  5 tiled image (A z-stacked image of 2629  ×  1636  ×  437 μm^3^) of the lateral olfactory tract and olfactory cortex, with mitral and tufted cell axons labeled with seven-color Tetbow. Adapted with permission from reference [108]. Copyright 2024 Nature communications.

Fluorescence imaging is widely used for the mesoscopic mapping of neuronal connectivity. However, neurite reconstruction is challenging, especially when neurons are densely labelled. Imai et al. report a strategy for the fully automated reconstruction of densely labelled neuronal circuits. Firstly, the stochastic super-multicolor labeling with up to seven different fluorescent proteins was established using the Tetbow method (Figure 9A) [108]. With this method, each neuron is labeled with a unique combination of fluorescent proteins, which are then imaged and separated by linear unmixing. The authors also establish an automated neurite reconstruction pipeline based on the quantitative analysis of multiple dyes (QDyeFinder), which identifies neurite fragments with similar color combinations (Figure 9B). The strategy allows the reconstruction of neurites for up to hundreds of neurons at the millimeter scale without using their physical continuity.

Although the Brainbow and Tetbow systems offer significant advantages in neural circuit labeling, they still have some limitations. First, these systems rely on genetic editing techniques, which may introduce unpredictable genomic changes that could affect the normal function of cells. Additionally, although the temporal control of the Tetbow system has been enhanced, challenges remain in precisely controlling the timing and accuracy of labeling. Furthermore, the efficiency of labeling and the stability of fluorescent signals may be impacted during long-term observations. To address these issues, future improvements could include optimizing the selection and expression strategies for Cre recombinase, integrating non-invasive labeling techniques, and using novel fluorescent proteins or molecular tools to enhance the sensitivity and stability of the system. Combining these methods with other neural circuit analysis techniques, such as virus-mediated labeling and single-cell RNA sequencing, could further improve the accuracy of labeling and the depth of data, providing more comprehensive insights into neuroscience research.

## 3. Labeling and Imaging of Cerebrovascular Networks

The cerebral vasculature is an anatomically and histologically inhomogeneous 3D traffic network for blood cells, nutrients, oxygen, metabolic wastes, signaling molecules, etc. [109,110,111]. Cerebrovascular imaging plays a pivotal role in elucidating the anatomical and functional organization of the brain’s vascular system. Structural and molecular analyses of cerebral vasculature provide fundamental insights into cerebral circulation and cerebrovascular diseases [112,113]. It is important to visualize entire vascular networks down to the capillary level in order to facilitate a better understanding of various vessel-associated diseases and to design targeted therapy. The visualization of the cerebrovascular network has evolved significantly over the past decades, driven by the need to understand the structural and functional basis of cerebral blood flow and neurovascular coupling [114]. Early studies utilized conventional histological staining and vascular casting methods, such as India ink perfusion and resin corrosion casting, to reveal the gross architecture of cerebral vessels [115].

**Figure 10:**
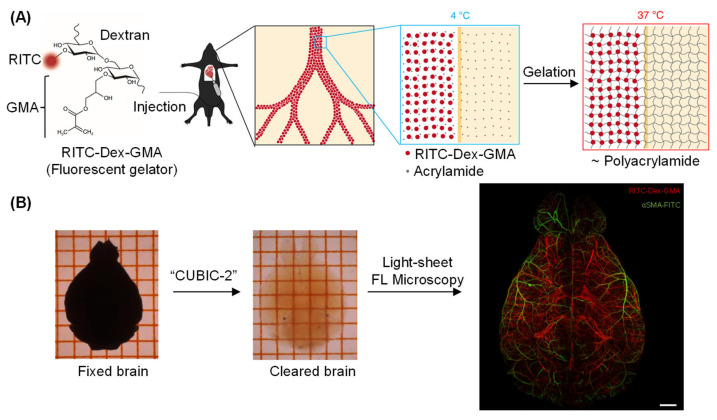
The SeeNet system for labeling and imaging of cerebrovascular networks. (**A**) Design of the BBB-impermeable fluorescent crosslinker RITC–Dex–GMA. (**B**) Maximum projection of light-sheet fluorescence microscopic images of the entire vasculature in a SeeNet-treated brain into a single stacked photo. Casted vessels are shown in red. Arterioles immunolabeled with anti-αSMA are shown in green. Scale bar: 1 mm. Adapted with permission from reference [121]. Copyright 2020 nature communications.

With the advent of fluorescence microscopy, vascular labeling transitioned towards the utilization of fluorescent dyes that could perfuse through the vascular lumen and delineate morphology at a micron-scale resolution [116,117,118]. Modern techniques combine these labeling strategies with advanced imaging modalities, such as confocal, two-photon, and light-sheet microscopy, as well as tissue-clearing approaches [119,120]. This combination has enabled deep-tissue visualization of vascular networks in three dimensions. SeeNet employed perfusion of a multifunctional BBB-impermeable crosslinker, vascular casting by temperature-controlled polymerization of hybrid hydrogels, and a bile salt-based tissue clearing technique (CUBIC-2) optimized for observation of vascular connectivity (Figure 10A) [121]. The technology has been demonstrated to facilitate near-complete 3D visualization of whole-brain cerebral vasculatures at the single-microvessel level, and unveil a hitherto unidentified vascular pathway that bridges cerebral and hippocampal vessels (Figure 10B). Recent innovations in the field have employed endogenous contrast mechanisms, such as autofluorescence and optical coherence tomography, to capture vascular morphology without the need for exogenous probes [122,123,124]. These developments have collectively transformed vascular imaging, moving from static, 2D visualization to dynamic 3D mapping across whole-brain volumes.

**Figure 11:**
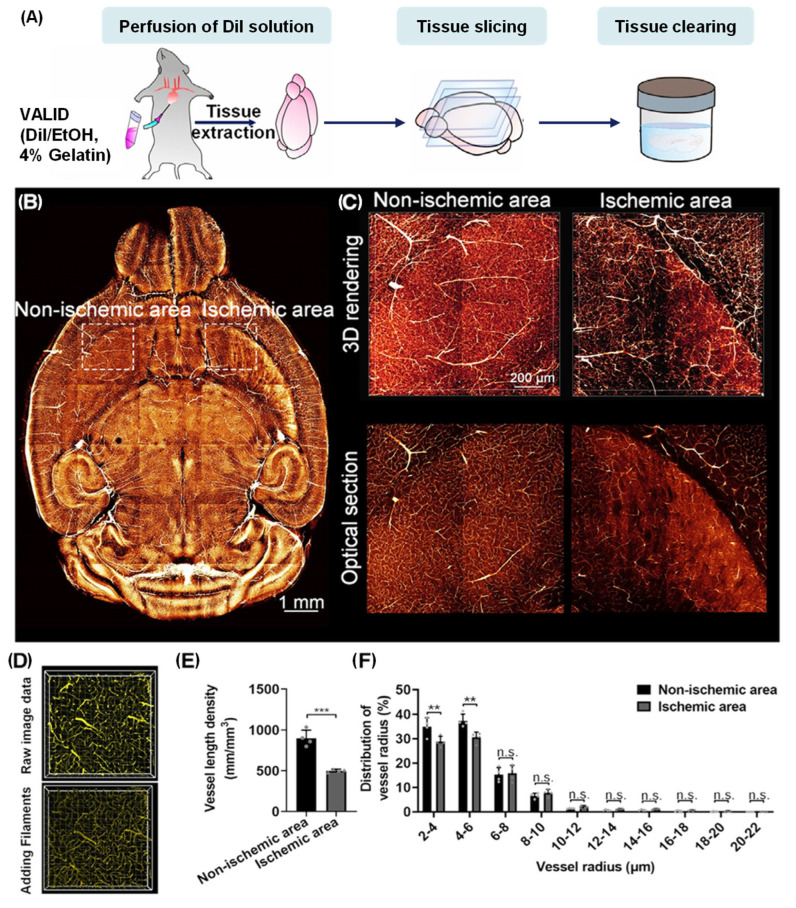
The VALID system for quantitative analysis of cerebrovascular networks. (**A**) Experimental workflow. (**B**) Reconstruction of the vascular architecture. (**C**) Magnification of non-ischemic and ischemic regions marked in (**B**). (**D**) Representative images of the reconstruction and analysis of blood vessels. (**E**) Quantification of vessel length densities in non-ischemic and ischemic areas (*n* = 4). (**F**) Statistical analysis of the distribution of vessel radius in non-ischemic and ischemic regions (*n* = 4). All values are presented as the mean ± SD. Statistical significance in (**E**,**F**) (*** *p* < 0.001; ** *p* < 0.01; n.s., not significant, *p* > 0.05) was assessed using an independent-sample t test. Adapted with permission from reference [129]. Copyright 2023 Cell Reports Methods.

Abnormal changes in vascular structures have been demonstrated to be closely associated with diseases, including stroke, diabetes, and Alzheimer’s disease [125,126,127]. Fluorescence-based vascular labeling combined with tissue clearing has enabled quantitative reconstruction of capillary networks. When integrated with light-sheet imaging, these methods provide submicron resolution across millimeter-scale brain regions, thus bridging the gap between cellular-level vascular details and mesoscale connectivity [128]. The VALID method provided an effective vessel-labeling protocol that can be used in conjunction with tissue clearing and light-sheet microscopy. In the vessel-labeling process, VALID employed a mixture solution of lipophilic Dil dye with gelatin protein in perfusion to construct fluorescent hydrogel in situ, thereby impeding aggregation-caused quenching (Figure 11A) [129]. This process resulted in complete and uniform vessel-labeling patterns, with high signal-to-background ratios (Figure 11B,C). The application of the 3D VALID visualization technique was successful in quantification of microvascular impairment (ischemic area) in a stroke mouse model of middle cerebral artery occlusion (Figure 11D–F). Further advances in the field, including genetically encoded vascular reporters and in vivo two-photon imaging, have enabled real-time monitoring of vascular remodeling, angiogenesis, and blood–brain barrier integrity under physiological and pathological conditions [130,131,132,133]. Multi-contrast imaging, which combines vascular labeling with neuronal or glial markers, facilitates a comprehensive analysis of the neurovascular unit, thereby offering insights into the coordination of vascular dynamics with neuronal activity [134]. These methods have significantly expanded the scope of cerebrovascular research, enabling system-level integration of vascular structure and brain function.

## 4. In Vivo Labeling and Monitoring Related to Neuronal Activity

**Figure 12:**
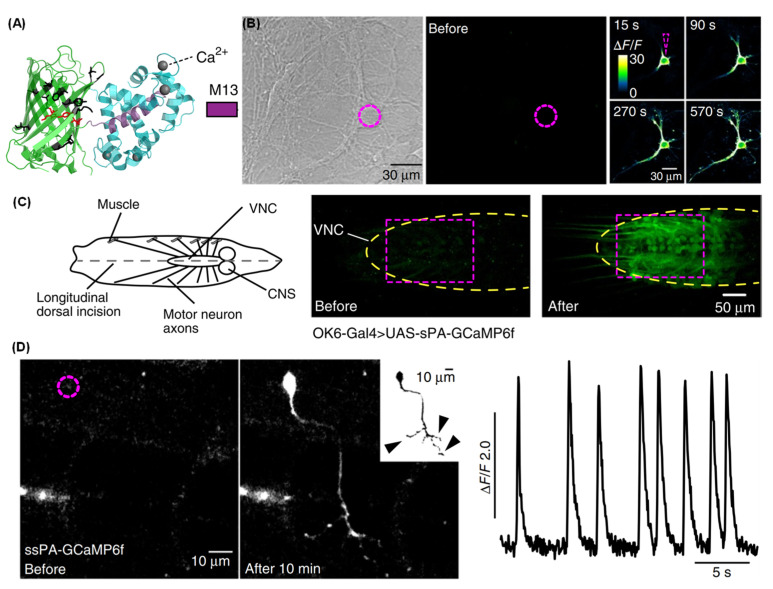
Fluorescent calcium imaging analysis using genetically encoded calcium indicators (GCaMP). (**A**) Cartoon of the calcium indicator (PDB: 3SG4), consisting of M13 (violet), a circularly permutated GFP (cpGFP, green) and calmodulin (CaM, cyan). (**B**–**D**) Visualization of single-cell calcium dynamics in cultured neurons (**B**), fruit flies (**C**), and zebrafish larvae (**D**). Adapted with permission from reference [142]. Copyright 2015 Nature Methods.

Calcium ions play a crucial role in brain neural activity. Imbalances in calcium ions may trigger neural damage and are closely associated with the onset of numerous neurological disorders. The advent of calcium imaging technology can be traced back to the 1980s, with the earliest fluorescent calcium indicators being synthetic molecule probes, such as Fura-2 [135]. These probes change their fluorescent properties upon binding to calcium ions, thereby indicating neuronal activity. With the advancement of genetic engineering, the emergence of genetically encoded calcium indicators (GCaMP) marked a new phase in calcium ion imaging [136]. GCaMP is a class of genetically encoded fluorescent proteins that provide real-time monitoring of neuronal activity. This technique combines fluorescence microscopy with molecular biology, enabling the dynamic observation of calcium signal changes in vivo at the single-cell level [137]. Calcium imaging was successfully applied to various aspects of research in animal models, including synaptic activity, neuronal population activity, and the connectivity and interaction between brain regions. By combining optogenetics and two-photon microscopy, researchers were able to monitor the functional activity of neural circuits at a larger scale, further revealing the dynamic regulatory mechanisms of the nervous system during behavioral tasks [138]. Through optimization of the fluorescence properties of GCaMP, calcium imaging techniques have enhanced the sensitivity of the signal and the ability to capture high-frequency activity [139]. Isacoff et al. have generated a family of photoactivatable GCaMPs [140,141] that combine attributes of high-contrast photolabeling with high-sensitivity Ca^2+^ detection in a single-color protein sensor (Figure 12A) [142]. They demonstrated the functionality of the indicators in cultured neurons (Figure 12B) as well as in fruit flies (Figure 12C) and zebrafish larvae (Figure 12D). The indicators enabled the visualization of morphology and high signal-to-noise measurements of activity, synaptic transmission and connectivity in single cells selected out of dense populations. The engineering strategy was transferable among different GCaMP versions, suggesting that this method will be applicable to future generations of sensors [143,144,145,146]. There are still several limitations to calcium imaging when it comes to monitoring neuronal activity [147]. Specifically, calcium signal changes are only indirectly related to membrane potential fluctuations, meaning calcium imaging does not fully represent actual firing patterns of neurons [148].

**Figure 13:**
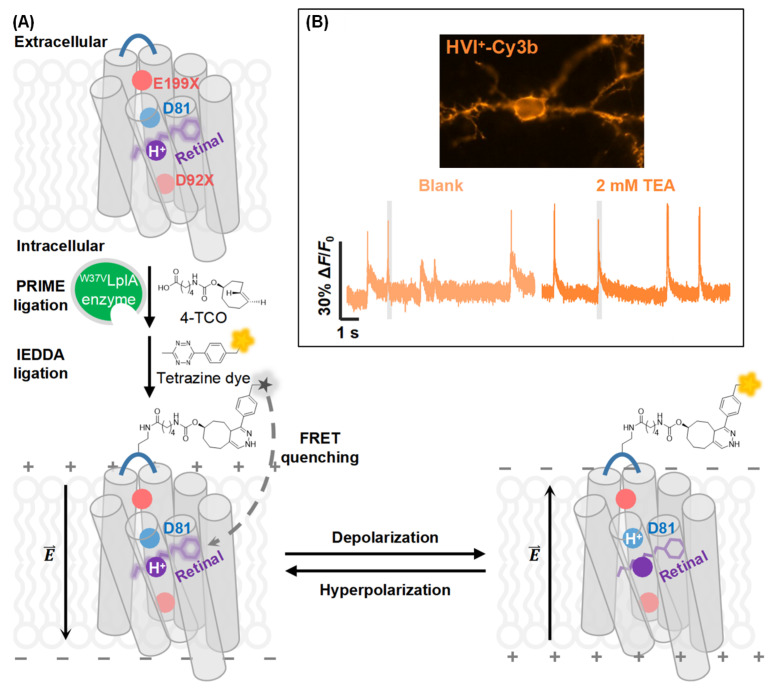
Design and application of the hybrid voltage indicator (HVI) system. (**A**) Scheme of the HVI assembly and its voltage sensing mechanism. (**B**) Epifluorescence voltage imaging of hippocampal neurons expressing HVI-Cy3b in the absence (blank) and the presence of 2 mM TEA (tetraethylammonium). Adapted with permission from reference [156]. Copyright 2025 Science Advances.

Membrane potential is a fundamental biophysical property of cells and is dynamically regulated by the actions of ion channels and pumps. In electrically excitable cells, including neurons, cardiomyocytes and pancreatic islet cells, their membrane potentials are intimately linked to cellular physiology, ranging from brain cognition and cardiac pacing to regulated hormone secretion [149,150,151]. Voltage-sensitive fluorescent indicators can emit fluorescent signals in response to changes in membrane potential [152]. The earliest applications of voltage-sensitive indicators are known to date back to the 1970s, including small-molecule dyes such as Di-8-ANEPPS and FluoVolt [153,154,155]. Voltage indicators have enabled the visualization of electrical activities at the single-cell level across large cell populations. In order to reduce background interference during voltage imaging, Zou et al. developed a smart positive-going hybrid voltage indicator (HVI) through the modulation of Förster resonance energy transfer (FRET) quenching via depolarization/hyperpolarization (Figure 13A) [156], thereby enabling accurate reporting of action potential waveforms in neurons expressing HVI-Cy3b (Figure 13B).

Voltage imaging has become a crucial tool for studying the dynamics of cell membrane potential, especially for real-time monitoring of neuronal firing activity. Through this technique, researchers were able to observe the spatial distribution of neuronal activity at the single-cell or even single-synapse level, as well as its relationship with neural circuits in live animals [157,158,159]. For example, the ‘Arclight’ indicating system has been widely used in the mouse cortex to monitor membrane potential changes, thereby revealing the connection between neuronal population activity and behavioral tasks [160]. The integration of two-photon microscopy and optogenetics has enabled the concurrent imaging of membrane potential and optical stimulation, thereby facilitating the elucidation of neural circuit functions [161,162]. Future research could focus on developing fluorescent dyes with superior optical performance and biocompatibility, while enhancing signal sensitivity and stability [163,164]. In comparison to calcium imaging, voltage imaging provides more direct membrane potential information and can be used complementarily to enhance the comprehensiveness and accuracy of neural activity monitoring.

**Figure 14:**
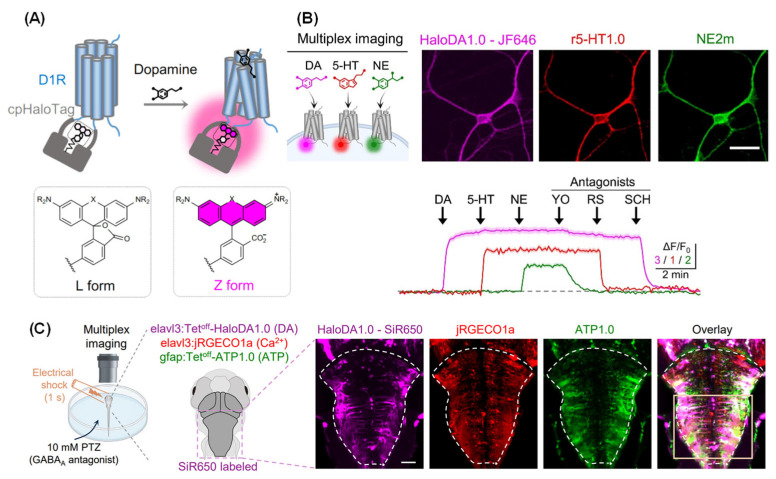
Development and imaging application of the dopamine sensor, HaloDA1.0. (**A**) Structure of the sensor. (**B**,**C**) Multiplex imaging in cultured neurons and zebrafish larvae. Scale bar: 50 μm. Adapted with permission from reference [168]. Copyright 2025 Science.

The spatial–temporal analysis of neuroactive molecules, including neurotransmitters, receptors and signaling molecules, has evolved from the integration of fluorescence-based imaging techniques [165]. Early studies primarily relied on immunofluorescence staining and fluorescence in situ hybridization (FISH) to map molecular distributions in fixed tissues. The advent of genetically encoded fluorescent proteins and chemical modification strategies for fluorescent probes has enabled real-time monitoring of these molecules in vivo. Single-molecule fluorescence imaging (e.g., smFISH and MINFLUX) approaches have enhanced detection sensitivity and spatial precision, thus allowing molecular dynamics to be visualized at the cellular level [166,167]. This class of labeling methods has been applied to elucidate the dynamic distribution and signaling of neuroactive molecules such as dopamine (DA), serotonin (5-HT), glutamate, and brain-derived neurotrophic factor (BDNF). For example, Li et al. developed a single-protein chemogenetic DA sensor, HaloDA1.0, which combines a cpHaloTag–chemical dye with the G protein-coupled receptor activation-based (GRAB) strategy, providing sub-second response kinetics and a far-red to near-infrared spectral range (Figure 14A) [168]. When used in conjunction with existing neuromodulator sensors, HaloDA1.0 demonstrated a high degree of versatility for multiplex imaging in cultured neurons (Figure 14B) and zebrafish larvae (Figure 14C), facilitating in-depth studies of dynamic neurochemical networks. The GRAB sensor series achieved millisecond-level temporal resolution for neurotransmitter tracking, providing insights into monoaminergic signaling alterations in models of depression and addiction. While calcium imaging reveals the activity patterns of neurons, neurochemical imaging has been shown to decipher the underlying regulatory mechanisms, and their integration holds promise for constructing a comprehensive map of brain function [169,170].

## 5. Neurodegenerative Structural Labeling

In the field of neurodegeneration research, fluorescent probes have a long-standing history in the labeling of pathological structures associated with neurodegenerative diseases [171]. Specifically, a range of small-molecule probes, including the representative Thioflavin T (ThT) [172,173], has been designed to visualize aberrant aggregates formed by amyloid-β (Aβ), tau, and α-synuclein [174,175,176,177,178,179,180]. The small molecular size and high diffusion capacity of these probes allows for rapid and uniform penetration of brain tissues, facilitating large-volume 3D reconstruction when combined with tissue clearing technologies [181]. Importantly, a few probes possess the ability to cross the blood–brain barrier, thereby capturing early pathological signatures suitable for disease diagnosis [182] Molecular probes engineered to selectively target oligomeric intermediates provided powerful tools for dissecting early aggregation events and evaluating anti-aggregation therapeutic efficacy [183]. Advancements in chemical probe design are driving the development of multi-target, cross-scale, and dynamic imaging approaches, transitioning their application from ex vivo staining toward in vivo monitoring of neurodegenerative pathology.

**Figure 15:**
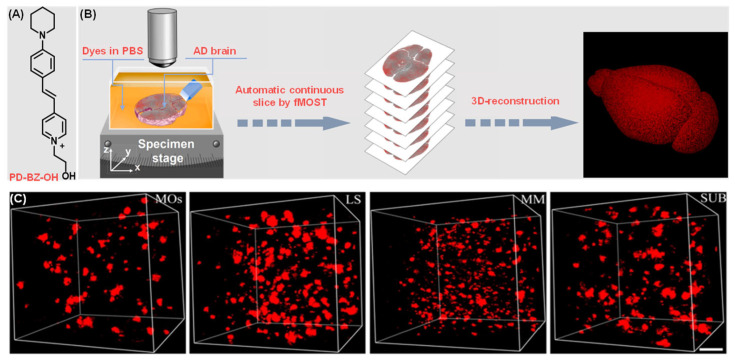
Automatic 3D mapping of Aβ plaques using AIE-active dye and the fluorescent micro-optical sectioning tomography (fMOST) system. (**A**) Structure of the water-soluble AIEgen, PD-BZ-OH. (**B**) Schematic workflow for 3D mapping of Aβ plaques with fMOST. (**C**) 3D-reconstructed image of Aβ plaques in the MOs (secondary motor area), LS (lateral septal nucleus), MM (medial mammillary nucleus) and SUB (subiculum) of a 5XFAD mouse, respectively. Scale bar: 50 μm. Adapted with permission from reference [187]. Copyright 2022 Chemical Engineering Journal.

Alzheimer’s disease (AD) is among the most prevalent degenerative diseases of the central nervous system [184]. The disease is pathologically characterized by the profusion of extracellular deposition of amyloid β-peptide in the form of β-amyloid (Aβ) plaques and vascular amyloid, involving the accumulation of amyloid fibrils [185]. The verification of Aβ plaque distribution in the brain is essential to obtain comprehensive information regarding the occurrence and development of AD [186]. In this regard, Zhu et al. reported a water-soluble AIE-active Aβ probe (named PD-BZ-OH) (Figure 15A) [187], which can label Aβ plaques in situ in a fluorogenic manner. This wash-free staining method was efficiently integrated into the fluorescent micro-optical sectioning tomography (fMOST) system for automatic 3D mapping of Aβ plaques in a mouse’s brain (Figure 15B). The spatial distribution, density, and morphology of Aβ plaques could be analyzed in great detail in 3D, as demonstrated in representative brain regions with dimensions of 200 × 200 × 200 μm (Figure 15C). This information would facilitate an exploration of the evaluation correlation between Aβ structure and neurodegenerative states on a brain-wide level.

Small-molecule probes usually exhibit insufficient binding specificity and conformational selectivity, leading to non-specific background signals and inadequate discrimination among aggregation stages. Compared with immunofluorescence and genetically encoded labels, small-molecule probes offer complementary strengths in early pathology identification, deep-tissue penetration, and translational potential, and are expected to continue serving as a strategic component in mechanistic investigation and diagnostic innovation for neurodegenerative diseases.

Immunofluorescence is widely employed for the specific detection of neurodegenerative structures, including Aβ, phosphorylated tau isoforms, and α-synuclein aggregates [188,189]. Immunofluorescence labeling relies on the high-affinity recognition of specific protein epitopes by antibodies and fluorophore conjugation for optical detection [190]. Fluorophores have evolved from FITC to new generations, such as Alexa Fluor and Cy dyes, which provide enhanced photostability and broader spectral coverage [191,192]. Meanwhile, signal amplification strategies, including tyramide signal amplification (TSA) and nanobody-based labeling, have improved detection sensitivity [193].

Multiplex immunofluorescence combined with confocal microscopy facilitates spatial mapping of amyloid deposition, synaptic degeneration, and neuroinflammatory responses [194]. In addition, the convergence of immunofluorescence with high-resolution microscopy techniques such as STED and SIM, as well as light-sheet imaging of cleared tissues, expands its capability for 3D structural reconstruction and pathological network topology analysis [195,196]. This elevates immunofluorescence as a versatile tool for dissecting region-specific vulnerability and cellular heterogeneity across neurodegenerative progression. The integration of tissue clearing techniques and automated slide-scanning systems facilitates large-scale and multi-layer pathological imaging of brain tissues, positioning immunofluorescence labeling as a cornerstone approach in both fundamental and diagnostic studies of neurodegenerative structural pathology.

**Figure 16:**
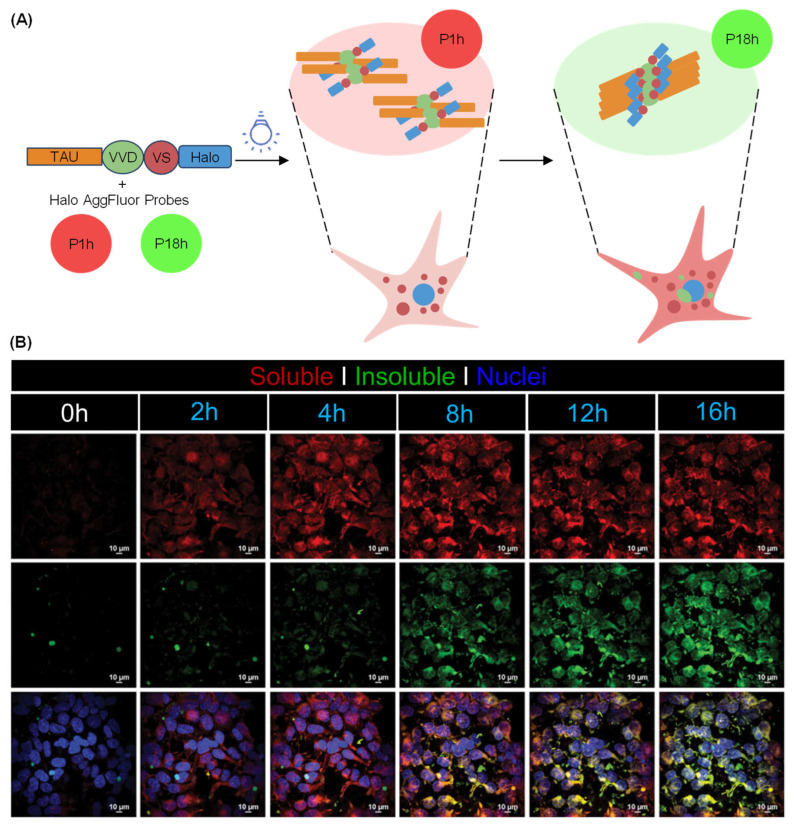
The optoTAU-Halo labeling system. (**A**) Schematic of the optoTAU-Halo model, and the viscosity-sensitive AggFluor dyes P1h (red; soluble oligomers) and P18h (green; insoluble aggregates). (**B**) Representative immunocytochemical analysis of soluble and insoluble tau species in cells. Scale bar: 10  µm. Adapted with permission from reference [202]. Copyright 2024 Communications Biology.

AD is a multifactorial neurodegenerative disease characterized by the accumulation of Aβ plaques and neurofibrillary tangles (NFTs) [197,198]. The NFT burden, but not the Aβ plaque load, more precisely correlates with the onset of cognitive impairment and regions of brain atrophy in AD patients [199,200,201]. Thathiah et al. developed a light-responsive tau protein (optoTAU) and used viscosity-sensitive AggFluor probes to investigate the consequences of tau aggregation in neurons and identify modifiers of tau aggregation in AD and other tauopathies (Figure 16A) [202]. Moreover, the optoTAU system reproduced the biological and structural properties of tau aggregation observed in human brains and the pathophysiological transition in tau solubility in live cells (Figure 16B). These findings have broad implications for the characterization of aggregation-prone proteins and investigation of the complex relationship between protein solubility, cellular function, and disease progression.

Despite its strengths in target specificity and labeling stability, immunofluorescence is still faced with challenges including limited antibody penetration, non-specific adsorption, reduced signal-to-noise ratio in deep-tissue imaging, and quantification variability caused by tissue fixation and processing. These limitations are particularly evident in large brain specimens, where slow antibody diffusion and epitope masking compromise the uniformity and depth of the labeling process. To enhance performance, ongoing efforts are centered on the following: antibody engineering (e.g., nanobodies and aptamers), intelligent fluorophore design, enhanced tissue clearing materials, and automated image-analysis workflows to optimize penetration and signal reliability [203,204,205]. When compared with small-molecule probes, immunofluorescence offers unique strengths in terms of precise pathological recognition and disease stratification. The two modalities offer complementary advantages across spatial–temporal scales and translational feasibility, which collectively underpin comprehensive mechanistic investigation and the development of multimodal diagnostic strategies for neurodegenerative disorders.

The core value of specific fluorescent labeling for Aβ plaques, neurofibrillary tangles, and α-synuclein aggregates extends far beyond pathological diagnosis. By integrating tissue clearing techniques with whole-brain imaging technologies (such as fMOST), researchers can accurately map the three-dimensional distribution of pathological proteins across the entire brain, revealing their patterns of propagation along specific neural circuits. This directly relates to the pathological remodeling of both macro- and micro-scale brain structural networks. Furthermore, probes designed to monitor soluble oligomers provide tools for in vivo investigation of early events such as synaptic loss and neuronal connectivity impairment, thereby linking molecular pathology with circuit dysfunction at a structural level. Therefore, neurodegenerative structural labeling serves not only as a tool for disease biomarker detection but also as a key technology for dissecting the dynamic evolution of brain structural networks throughout disease progression.

## 6. Targeted Imaging of Brain Tumors

Tumor-specific fluorescent probes serve as crucial tools for precise tumor localization and surgical navigation [206]. Since the 1980s, fluorescent probes have gradually emerged in the field of tumor imaging [207]. With the combination of fluorophores (such as nanoparticle fluorescent probes) and targeting moieties (such as monoclonal antibodies), the targeting ability, stability and optical performance of these tumor-specific probes have been significantly enhanced [208,209]. In brain tumor targeting imaging, tumor-specific fluorescent probes effectively enhance the spatial boundary resolution between tumor tissue and surrounding normal brain tissue by targeting brain tumor-specific markers (such as EGFRvIII mutation and GD2 ganglioside) [210,211].

Fluorescence-guided tumor resection has been applied in real-time navigation during surgeries for gliomas and malignant brain tumors [212]. Compared with traditional MRI or CT techniques, fluorescence imaging offers higher spatial resolution and more convenient real-time imaging. Recent applications of near-infrared (NIR) indocyanine green probes in conjugation with surgical navigation systems have yielded real-time visualization of malignant brain tumors, thereby assisting surgeons in accurately delineating tumor boundaries and minimizing damage to normal tissue [213,214]. The integration of nanoparticle probes and targeted drug delivery systems not only facilitates imaging functionality but also enables direct delivery of anti-tumor drugs to the tumor site [215,216]. The combination of tumor-specific fluorescent probes with photodynamic therapy has been shown to enhance the efficacy of local tumor drugs and control the postoperative recurrence rate [217].

**Figure 17:**
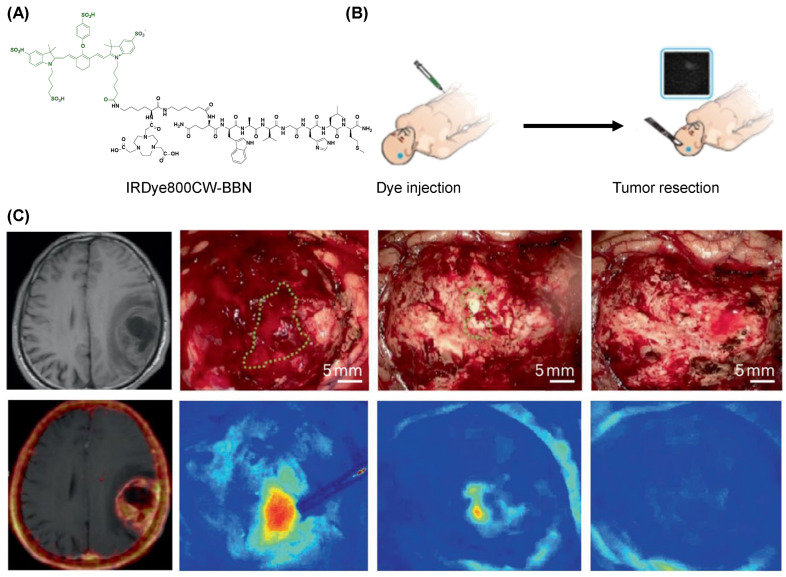
Fluorescence-guided tumor resection of glioblastoma multiforme (GBM). (**A**) Chemical structure of IRDye800CW (green) with the BBN (black). (**B**) Enrollment and research scheme for the guided GBM patients. (**C**) ^68^Ga-IRDye800-BBN PET/NIRF dual-modality imaging resection for patients. Adapted with permission from reference [218]. Copyright 2018 Theranostics.

Despite the use of fluorescence-guided surgery, achieving maximum safe resection of glioblastoma multiforme (GBM) remains a significant challenge. This has imposed limitations on surgeons, confining them within the timeframe between preoperative diagnosis and intraoperative treatment. Chen et al. developed a unique dual-modality PET/optical imaging probe based on the ^68^Ga-BBN PET tracer to specifically image the in vivo expression of the GRPR receptor on gliomas of different World Health Organization grades (Figure 17A) [218]. This was the first translational study performed in GBM patients comparing the preoperative GRPR receptor biodistribution with the traditional gadolinium enhancement in MRI (Figure 17B). This unique dual-modality imaging probe enabled preoperative assessment for resection accuracy and real-time optical navigation intraoperatively for achieving maximal safe resection (Figure 17C). Through interdisciplinary technology integration, the development and application of tumor-specific fluorescent probes will not only help advance precision medicine for brain tumors but also provide new directions for early diagnosis and precise treatment of other types of cancer.

Although fluorescence-guided tumor resection is a clinically oriented application, the underlying technology profoundly serves the study of brain structure. Firstly, probes targeting tumor-specific antigens such as EGFRvIII and GD2 can delineate the structural boundaries between tumor and normal brain tissue at a cellular scale—a resolution difficult to achieve with traditional MRI—thus providing high-resolution mapping of the structural damage inflicted by tumor invasion on the surrounding neurovascular unit (blood–brain barrier, neurons, and glial cells). Secondly, the design principles for developing tumor probes capable of crossing the blood–brain barrier—such as size, charge, and ligand selection—are equally applicable to designing specific in vivo markers for neuronal or glial cell subtypes. Finally, the need for precise identification of tumor boundaries during surgical navigation drives the advancement of real-time, high-resolution, multispectral imaging technologies. These technological platforms can, in turn, be repurposed for dynamic structural observations of the healthy brain. Therefore, the technical challenges and innovations in the field of brain tumor imaging constitute a significant driving force for methodological progress in in vivo, high-precision brain structure research.

Current fluorescence imaging techniques still rely on manual real-time operation during surgery [219]. Intelligent image analysis technologies, such as AI-based image processing and automated navigation systems, are gradually being integrated with fluorescence imaging techniques to improve the precision and reliability of tumor resection [220]. In the future, the combination of tumor-specific fluorescent probes with different imaging module techniques (such as MRI and CT) and therapeutic methods (such as radiotherapy and photodynamic therapy) will provide innovative solutions for the precise treatment and prognostic evaluation of brain tumors.

## 7. Conclusions and Outlook

The brain is a complicated dynamic system of large volume, which requires high spatial–temporal resolution in analysis for reliable interpretation. Fluorescent labeling techniques have been utilized to facilitate multiscale visualization of brain structures, ranging from the microscopic to the macroscopic level. This objective is realized through the implementation of specific labeling methodologies that target neurons, blood vessels, synapses, and other components. Recent advancements in tissue clearing methods, coupled with deep imaging technologies, have emerged as pivotal solutions to overcome these imaging barriers, allowing for high-resolution, volumetric analysis of intact brain tissues. This approach has resulted in significant insights into the mechanisms of brain function and the treatment of neurological disorders.

Fluorescent labeling technology has evolved from a mere tool for cellular visualization into a core engine for analyzing the multiscale structure and dynamics of the brain. This review systematically synthesizes labeling strategies ranging from microscopic synapses to whole-brain circuits, revealing a clear paradigm shift: from static, single-modal observation toward an era of dynamic, multifunctional integration and intelligent interpretation. However, this progress has also exposed fundamental technological bottlenecks and interdisciplinary challenges. Future breakthroughs will no longer rely solely on brighter fluorescence or higher resolution, but rather on how to systematically integrate molecular specificity, spatiotemporal scale, and biocompatibility, ultimately serving the mechanistic understanding of brain function and disease.

The future development of fluorescence labeling methods for brain structure research will rely on the synergistic advancement of novel fluorescent probes, cutting-edge imaging technologies, and multimodal data integration. These technological advancements are poised to facilitate high-resolution, dynamic imaging at the whole-brain scale, thereby offering deeper insights into the intricacies of neuroscience. The potential applications of this technology are manifold and include, but are not limited to, research into neurodegenerative diseases, brain network mapping, precise neural modulation, brain–computer interfaces, and drug development. The technology offers revolutionary support for understanding brain complexity, treating neurological disorders, and developing intelligent neural technologies. Achieving these objectives will be contingent on the establishment of collaborative frameworks between the domains of neuroscience, imaging technology, data science and engineering, with a view to addressing technological challenges and expediting the translation of research findings into practical applications.

## Figures and Tables

**Table 1:** Key feature comparison of major fluorescent labeling methods in neuroscience.

Labeling Methods	Key Advantages	Main Limitations	Application
**Small molecule** **labeling**	Applicable to different samples Real-time functional imaging Modulable in fluorescence With tissue clearing methods	Dye leakage and photobleaching Invasive methods Lack specificity	Circuit tracing (e.g., DiI/DiO)
**Immunolabeling**	High target specificity Multiplexable labeling	Fixation required Limited penetration depth Cross-reactivity risks	Antibody-based (NeuN, etc.)
**Viral-mediated** **labeling**	Strong trans-synaptic specificity Genetic encodability Long-term expression	Variable tropism Ectopic expression Size limitations Temporal delay	Projection mapping (e.g., AAV1, HSV)
**Transgenic labeling**	Multicolor stochastic labeling Genetically stable and heritable Cell-type or region targeting possible Genetic precision	Color discrimination challenges Overexpression artifact limitations Time and cost	Connectomic barcoding: (e.g., Brainbow/Tetbow)

## Data Availability

The data presented in this study are available on request from the corresponding author.
